# Enhancement of NK Cell Cytotoxicity Induced by Long-Term Living in Negatively Charged-Particle Dominant Indoor Air-Conditions

**DOI:** 10.1371/journal.pone.0132373

**Published:** 2015-07-14

**Authors:** Yasumitsu Nishimura, Kazuaki Takahashi, Akinori Mase, Muneo Kotani, Kazuhisa Ami, Megumi Maeda, Takashi Shirahama, Suni Lee, Hidenori Matsuzaki, Naoko Kumagai-Takei, Kei Yoshitome, Takemi Otsuki

**Affiliations:** 1 Department of Hygiene, Kawasaki Medical School, 577 Mastushima, Kurashiki, Okayama, 701–0192, Japan; 2 Comprehensive Housing R&D Institute, SEKISUI HOUSE, Ltd., 6-6-4 Kabutodai, Kizugawa, Kyoto, 619–0224, Japan; 3 Sumitomo Riko Co. Ltd., 1 Higashi 3-chome, Komaki, Aichi, 485–8550 Japan; 4 Yamada SXL Home Co. Ltd., 5F OAP Tower, 1-8-30 Tenma-bashi, Kita-Ku, Osaka, 530–0043, Japan; 5 Department of Biofunctional Chemistry, Division of Bioscience, Okayama University Graduate School of Natural Science and Technology, 1-1-1 Tsushima-Naka, Okayama, 700–0082, Japan; 6 Artech Kohboh, Co. Ltd., 57–29, Hattandago, Higashisonogi, Higashisonogi, Nagasaki, 859–3922, Japan; University of Manitoba, CANADA

## Abstract

Investigation of house conditions that promote health revealed that negatively charged-particle dominant indoor air-conditions (NCPDIAC) induced immune stimulation. Negatively charged air-conditions were established using a fine charcoal powder on walls and ceilings and utilizing forced negatively charged particles (approximate diameter: 20 nm) dominant in indoor air-conditions created by applying an electric voltage (72 V) between the backside of the walls and the ground. We reported previously that these conditions induced a slight and significant increase of interleukin-2 during a 2.5-h stay and an increase of NK cell cytotoxicity when examining human subjects after a two-week night stay under these conditions. In the present study, seven healthy volunteers had a device installed to create NCPDIAC in the living or sleeping rooms of their own homes. Every three months the volunteers then turned the NCPDIAC device on or off. A total of 16 ON and 13 OFF trials were conducted and their biological effects were analyzed. NK activity increased during ON trials and decreased during OFF trials, although no other adverse effects were found. In addition, there were slight increases of epidermal growth factor (EGF) during ON trials. Furthermore, a comparison of the cytokine status between ON and OFF trials showed that basic immune status was stimulated slightly during ON trials under NCPIADC. Our overall findings indicate that the NCPDIAC device caused activation of NK activity and stimulated immune status, particularly only on NK activity, and therefore could be set in the home or office buildings.

## Introduction

Improvement of indoor air-conditions is very important in promoting human health because various illnesses may occur that impair the psycho-neuro-endocrino-immune (PNEI) status such as sick building syndrome (SBS) [1,2] and multiple chemical sensitivity (MCS) [3,4]. In addition, many microorganisms in the indoor environment also affect human health by causing infections and allergic reactions. SBS is caused by various chemicals and is typically associated with formaldehyde and volatile organic compounds (VOC) such as toluene, xylene and ethyl acetate [5,6]. Patients with SBS exhibit various symptoms such as mucous membrane irritation involving the eyes, nose and throat, headaches, fatigue, irritability, immunological disorders such as asthma and urticaria, as well as asthma-like symptoms (chest tightness and wheezing) and skin dryness and irritation [1,2]. Although some practices such as the questionnaire QEESI (Quick Environment Exposure Sensitivity Inventory) [7,8] and the digital self-examination designated “Chemiless Necessity Test” produced by the Chemiless Town project of the Center for Preventive Medical Science, Chiba University have been implemented to identify people with SBS [9–11], practical treatments for SBS have not been established and avoiding exposure to chemicals and places that induce symptoms is the only on-target method to assist affected individuals [1,2]. Furthermore, the situation involving MCS patients is much more difficult because these patients have specific sensitivity against various chemicals [3,4]. In addition to these factors, microorganisms such as fungus, bacteria and mites also affect human health through their infectious diseases and biological components such as spores and epidermal debris that act as allergens [12,13].

**Fig 1:**
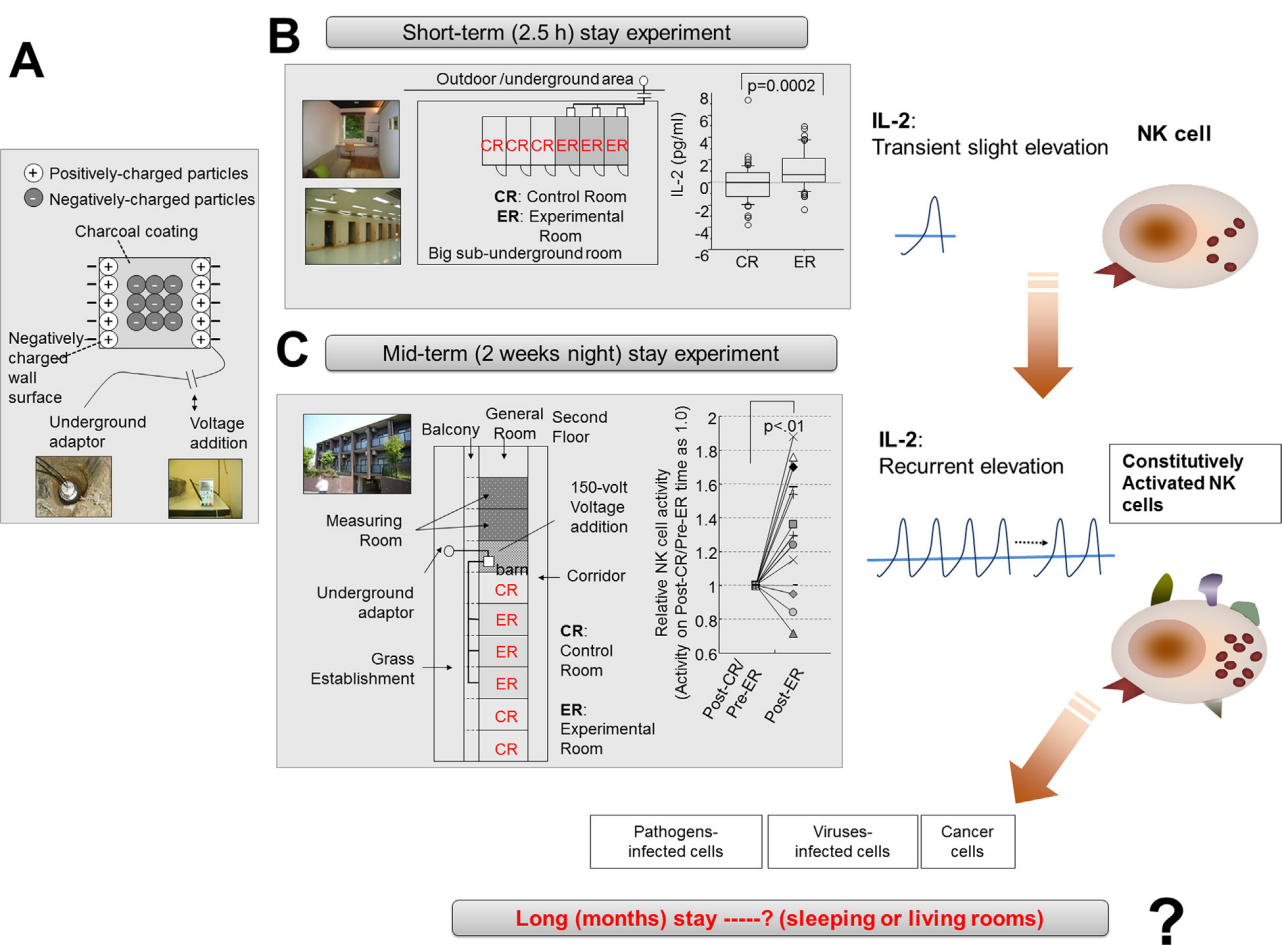
Schematic summary of previous experiments and development of negatively charged-particle dominant indoor air-conditions (NCPDIAC). (A) NCPIADC was established by painting a charcoal coating made by fine charcoal powder onto the wall and ceiling of a room. The charcoal coating designated as Health Coat was produced by Artech Kohboh Co. Ltd. In addition, a forced negatively charged-particle dominant indoor air condition was created by applying an electric voltage (72 volts) between the backside of the walls of a room and the ground. (B) The 2.5-hour stay experiments were performed using small rooms with or without NCPDIAC conditions. Sixty healthy volunteers were admitted to the rooms and biological monitoring was initiated regarding common blood testing, measures for the autonomic nerve system, and immunological parameters such as cytokines. Results revealed a small but significant elevation of interleukin (IL)-2 after entering NCPDIAC rooms. (C) Night-stay experiments over a period of 2 weeks were conducted with volunteers staying in a dormitory fitted with the NCPDIAC devices, and various biological parameters were monitored including those involved in the short-term experiments, as well as NK cell activity, since these parameters may be altered after two weeks. Results showed that NK cell activity was the only parameter exhibiting a significant change. Schematic biological changes caused by NCPDIAC is also presented in the right side of figure. NCPDIAC caused a slight but certain increase of IL-2 during short-term and mid- to long-term (weeks to months) exposure periods and induced NK cell killing activities due to the recurrent and accumulated elevation of IL-2.

To improve the quality of indoor air-conditions, many custom-house building companies have been trying to establish health protecting and promoting indoor conditions in relation to temperature, humidity, airtightness, and air streaming [14,15]. In keeping with this purpose, we have been investigating the beneficial health effects of establishing negatively charged-particle dominant indoor air-conditions [16,17]. The particles are approximately 20 nm diameter. The condition was created by painting a charcoal coating made using fine charcoal powder onto the wall and ceiling of a room. The charcoal coating designated as Health Coat was produced by Artech Kohboh Co. Ltd. [18]. In addition, a forced negatively charged-particle dominant indoor air condition was created by applying an electric voltage (72 volts) between the backside of the walls of a room and the ground as shown in Fig 1A. These mixed devices were designated SUMICAS and provided by Yamada SXL Home Co. Ltd. It is known that a high negatively charged-particle dominant air condition is present in a forest, particularly near a waterfall. In addition to improving the emotional moods of humans exposed to a forest atmosphere, the immunological effects of a forest tour (forest bathing) over a period of a few days have been reported to include increased NK cell activity [19,20]. In the forest environment, many other factors such as air-conditions, scents from plants, the sounds of a babbling stream and small birds, as well as the colors of nature may affect the PNEI status of humans. It is on the basis of these observations that the biological effects of negatively charged-particle dominant indoor air-conditions (NCPDIAC) have been examined [16,17].

As shown in Fig 1B and 1C, two trials were performed. Initially, an experiment involving a 2.5-hour stay was conducted using small rooms with or without NCPDIAC conditions. Sixty healthy volunteers were admitted to the rooms and biological monitoring was initiated regarding common blood testing, measures for the autonomic nerve system, and immunological parameters such as cytokines. Results revealed a small but significant elevation of interleukin (IL)-2 after entering NCPDIAC rooms as shown in the right panel of Fig 1B [16]. Night-stay experiments over a period of 2 weeks were conducted with volunteers staying in a dormitory fitted with the NCPDIAC devices (Fig 1C), and various biological parameters, including those involved in the short-term experiments, were monitored and included NK cell activity and urine concentration of 8-hydroxy-2'-deoxyguanosine (8-OHdG) as a maker for oxidative stress, since these parameters may be altered after a two-week stay in the experimental rooms. The results of these experiments indicated that the only significant changes involved NK cell activity, as shown in the right panel of Fig 1C [17].

Examination of these two experimental periods involving a short-term and mid-term stay in NCPDIAC is shown in Fig 1B and 1C. The accumulation of the short-term, transient and recurrent elevation of IL-2 affects NK cells and resulted in an increase of their activity. Therefore, living under NCPDIAC for periods of months and years at night time while sleeping or in the living room may increase basic NK cell activity, and these conditions may help people by reducing the level of cancerous diseases and severe infectious diseases from viruses and bacteria.

In response to these findings concerning short-term and mid-term experiments, we have been conducting monthly stay experiments in NCPDIAC rooms. Seven volunteers were recruited and required to switch a SUMICAS device on or off every three months to respectively promote or remove NCPDIAC. Biological monitoring in these experiments focused particularly on NK activity and various changes in immunological cytokines.

## Materials and Methods

### Subjects and ethical issues

All seven healthy volunteers are Japanese living in Japan and were asked to join this project by first-class registered architects who are colleagues of the authors. The average age of volunteers was 54.86 ± 9.15 years and included five males and two females. All volunteers built or re-constructed their residential home before being recruited to this project and agreed to set a SUMICAS device for experiments. The SUMICAS devices were provided by Artech Kohboh Ltd. and Yamada SXL Home Co. Ltd. The experiment was approved by the ethical committee of Kawasaki Medical School and associated hospital (#854), and written informed consent was obtained from all volunteers before starting experiments.

### SUMICAS setting and biological monitoring

**Fig 2:**
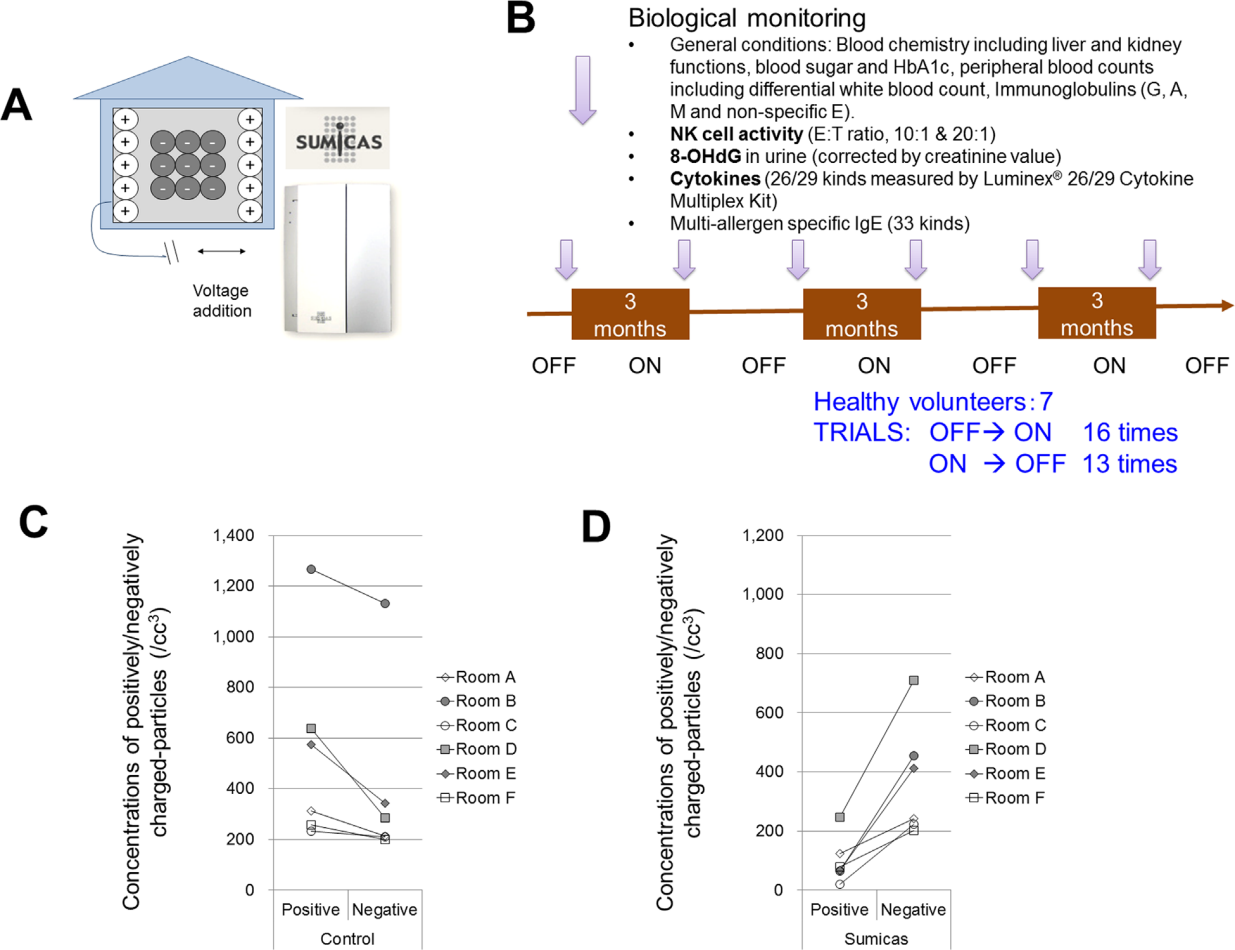
The SUMICAS setting for NCPDIAC was done by Artech Kohboh Ltd. and Yamada SXL Home Co. Ltd. The appearance of the SUMICAS device is shown panel A, and has an approximate size of 30(H) x 20 (W) x 4(D) cm. Volunteers switched the device ON or OFF by themselves every three months when blood and urine samples were obtained for biological monitoring (B). After pre-sampling for blood and urine, all volunteers turned SUMICAS ON and then switched SUMICAS either OFF or ON every three months. A total of 16 OFF to ON trials (On trial) have been safely conducted so far, as well as 13 OFF to ON trials (Off trial). The longest two volunteers completed 4 ON trials and 3 OFF trials over a period of one year and nine months. The shortest volunteer completed only 2 ON trials and 1 OFF trial because he was subsequently transferred to another location by his employer. The positively and negatively charged particles in the representative six rooms at where SUMICAS apparatus was set were measured using as Ion Counter (EM-1000, Eco Holistic Inc., Suita, Japan) during OFF period (indicated as control, panel C) and ON period (indicated as Sumicas, panel D).

The SUMICAS devices were provided by Artech Kohboh Ltd. and Yamada SXL Home Co. Ltd. The appearance of a SUMICAS device is shown in Figs 1A and 2A and has an approximate size of 30(H) x 20(W) x 4(D) cm. Volunteers switched the device ON or OFF by themselves every three months when blood and urine samples were obtained for biological monitoring. All seven volunteers were not stayed in SUMICAS condition in the day-time, when they were working. They only lived in SUMICAS at the night time.

The parameters investigated for biological monitoring were as follows (Fig 2B):

1. General condition: blood chemistry including liver (AST, ALT and γGTP) and kidney (BUN and creatinine) functions, blood sugar, HbA1C, peripheral blood counts, including differential white blood cell count.
2. Immunoglobulins (IgG, A, M and non-specific IgE)
3. NK activity (against a k562 human leukemia cell line; NK: target cell ratios were 10:1 and 20:1, as reported previously [17, 21].
4. 8-OHdG in temporary urine (corrected by creatinine concentration) [22,23]
5. Multi-allergen specific Ig E (33 kinds)
6. Cytokines (26 kinds measured by Luminex 26 Cytokine Plex Kit Human Cytokine/Chemokine Panel 1 (MPXHCYTO60KPMX26, Merck Millipore, Billerica, MA) for initial five samples; other samples were measured using 29 kinds of cytokines (HCYTMAG-60K-PX29, Merck Millipore).

Items 1) to 5) were performed by BML (Bio Medical Laboratories) Inc., Tokyo, Japan), and item 6) was performed by the Department of Hygiene, Kawasaki Medical School, Kurashiki, Japan.

After pre-sampling for blood and urine, all volunteers turned SUMICAS ON and then switched SUMICAS either OFF or ON every three months. A total of 16 OFF to ON trials (On trial) have been safely conducted so far, as well as 13 OFF to ON trials (Off trial). The longest two volunteers completed 3 ON trials and 3 OFF trials over a period of one year and nine months. The shortest volunteer completed only 1 ON trials and 1 OFF trial because he was subsequently transferred to another location by his employer.

Regarding investigation of NK activity, the change from OFF to three months of ON and then ON to OFF was compared with the actual NK activity (%) and the change of the ratio while setting the NK activity before ON or OFF setting as 1.0 to standardize individual variation.

For the examination of cytokines, three kinds of data were analyzed: (1) the actual concentration (pg/ml) in plasma, (2) the logarithmic value of the actual concentration, since elevation of cytokines often occurs according to the logarithmic range, and (3) the altering ratio of the logarithmic value since the concentrations of cytokines vary from volunteer to volunteer and the ratio using a pre-data value of 1.0 may support and standardize the recorded alteration of cytokines. The alteration of cytokines was analyzed by considering ON trials as the paired samples (in the same volunteer), and OFF trials as the paired samples (in the same volunteer). In addition, the concentration of cytokines in the ON and OFF trials (the latter concentration subtracted by the former data) was investigated to determine the elevation of cytokines during each trial period. A comparison of the cytokine elevation levels was therefore performed between ON and OFF trials.

### Particles concentrations

The positively and negatively charged particles in the representative six rooms at where SUMICAS apparatus was set were measured as reported previously [16,17], using as Ion Counter (EM-1000, Eco Holistic Inc., Suita, Japan).

### Statistical analyses

Comparisons of NK activities and concentrations of cytokines (actual data, logarithmic value, and ratio of logarithmic values) between ON or OFF trials were performed using Student’s t-test (paired sample, double-sided), as was comparison of the elevated values of each cytokine during ON and OFF trial periods. In addition, to extract the cytokines of interest in the ON and OFF trials, we performed multi-regression analysis and the stepwise regression test by setting the ON trial as 10.0 and the OFF trial as 1.0.

In each analysis, a p value below 0.05 was considered significant and a value between 0.05 and 0.1 was considered to possess tendency. All statistical analyses were performed using SPSS ver. 22 (IBM Japan, Tokyo, Japan).

## Results

### Procession of experiments

The essential aim of this study was to determine whether NK cell activity was enhanced during a month-long stay in NCPDIAC, since short-term (2.5 hours) and mid-term (two-week night stay) experiments indicated that the temporary accumulation and transient increase of IL-2 during short-term exposure to NCPDIAC induced enhancement of NK cell activity. In addition, we aimed to develop the NCPDIAC apparatus so that it could be applied to single-family houses and/or multi-unit apartment buildings for prolonged periods spanning months to years in order to improve the health of residents.

As shown in Fig 2C and 2D, all the rooms in which SUMICAS was set showed slightly higher positively charged-particles in the control (SUMICAS OFF) conditions (Fig 2C) and negatively charged particles higher condition in the SUMICAS ON conditions (Fig 2D).

In this study, NCPDIAC was applied to actual independent personal houses and units in multi-apartment buildings. There was no adverse effect during the ON trial periods for any of the volunteers, and no abnormal data among the clinical parameters were measured just after the ON or OFF trials. In addition, no subjective symptom was caused by NCPDIAC. Therefore, it would be safe to install this NCPDIAC device in personal houses.

### NK activity

**Fig 3:**
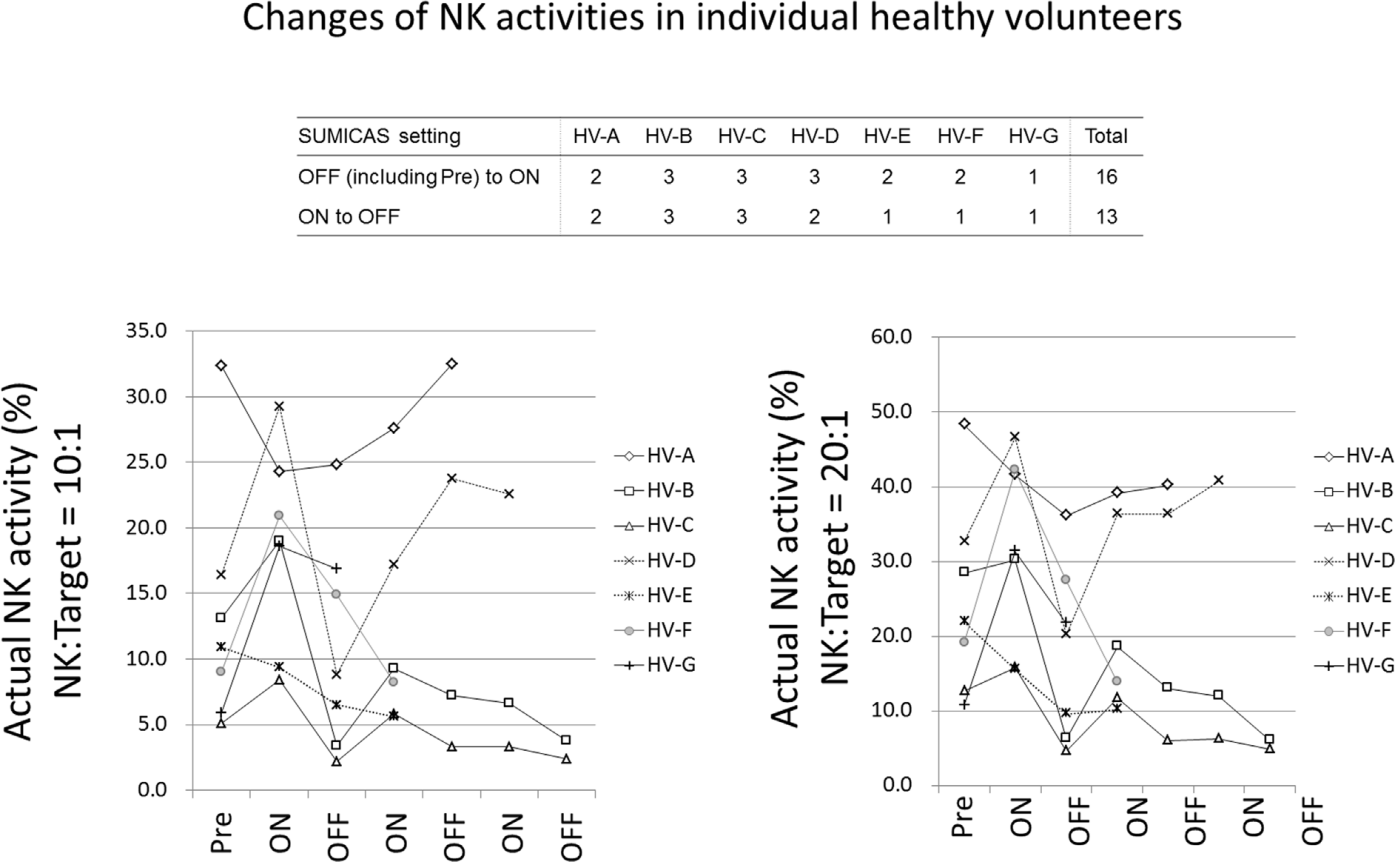
Changes of NK activities in individual healthy Volunteers. Each volunteer experienced ON and OFF trials of SUMICAS setting as indicated. The active (%) NK activities in individual volunteers were plotted when the NK activities were measured as NK and Target cells in 10:1 (the left panels) and 20:1 (the right panels). The “ON” showed the data obtaining after living in three months ON period and “OFF” indicated the data measuring after living in three months “OFF” period.

The most important parameter was NK cell activity. As shown in Fig 3, among seven volunteers, three experienced OFF (including “PRE”) to ON, 3 times and ON to OFF, 2 times. One volunteer had 3 and 2 times, one had 2 and 2 times, two had 2 and 1 and one had 1 and 1 time(s) experiments for OFF (including Pre) to ON, and ON to OFF setting, respectively. Thus, in total, OFF (including initial Pre to ON) to ON, 16 times and OFF to ON, 13 times. Fig 3 revealed individual changes of NK activities (actual %) in 10:1 and 20:1 NK vs target cell ratios. In addition, there is no tendency in changes of NK cell activities by seasons. Moreover, although all volunteers switched SUMICAS device ON or OFF by themselves, there were no evidence or impressions that self-switching affected the results.

**Fig 4:**
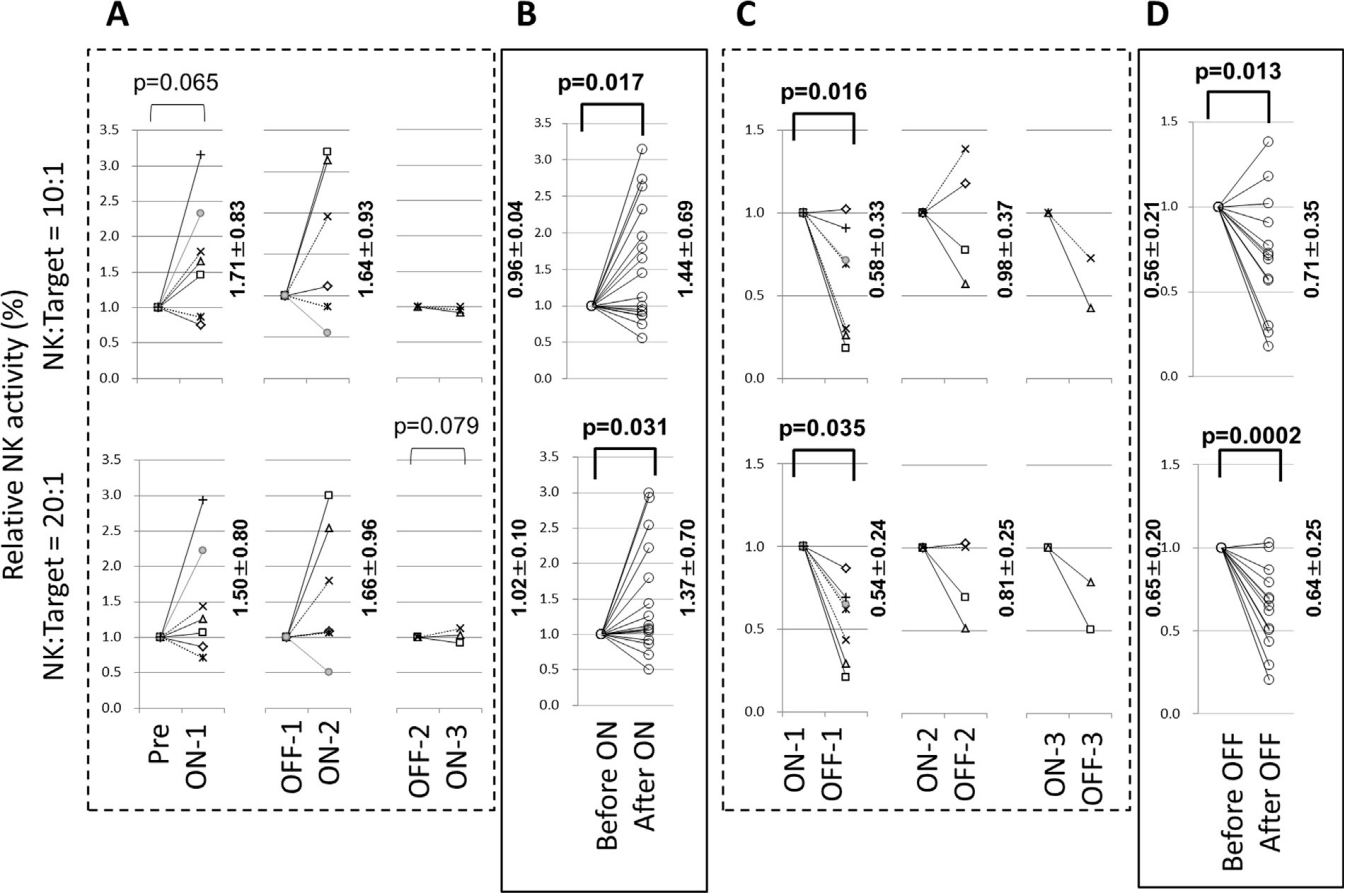
Relative changes of NK activities (setting before data as being 1.0 in each ON periods (including initial Pre to ON) in the initial, second and the third trials were shown in A. The upper panel showed ET ratio as 10:1 and lower panel showed 20:1. In panel B, total 16 ON trials were mixed and revealed. In panel C and D showed similar analyses during total 13 OFF trails.

As shown in Fig 4A, individual changes of relative NK activities revealing before data as 1.0 in each three months OFF to ON and ON to OFF trials in the first, second and third trials were shown. Among these analyses, only the first changes in 10:1 ET ratio showed the tendency of increase of NK activity. However, mix together all 16 OFF to ON trials, there were significant increase of changes of NK relative activities in 10:1 and 20:1 ET ratios (shown in Fig 4B).

Contrast to these OFF (including Pre) to ON trials, among the first, second and third ON to OFF trials, both 10:1 and 20:1 ET ratios showed significant decrease of relative NK activity as shown in Fig 4C. In addition, total 13 trials of ON to OFF changes showed significant decrease of relative NK activity in both ET ratios as shown in Fig 4D.

In addition, the changes of actual NK activities of total 16 OFF (including Pre) to ON and ON to OFF in 10:a or 20:1 ET ratios showed increasing tendencies on OFF to ON in both ET ratios and decreasing tendency on ON to OFF in 10:1 ET ratio. The ON to OFF trial in 20:1 ET ratio showed significant reduction of actual NK activity as shown in S1 Fig.

These results confirm that NCPDIAC over several months induced human NK activity, and is consistent with our previous reports showing that hour-long short-term and week-long mid-term exposure to NCPDIAC resulted in the accumulation and elevation of IL-2 and subsequent activation of NK cell activity [16,17].

### Alteration of cytokine production during ON and OFF trials of NCPDIAC

#### 1. Changes of actual concentrations of cytokines

Plasma samples obtained for the 16 ON and 13 OFF trials were used to measure the actual concentrations of various cytokines, Log_10_ concentrations, and ratio of Log_10_ concentrations (value of before data being 1.0) using a Luminex 26 or 29 Cytokine Plex Kit Human Cytokine/Chemokine Panel. Statistical differences were analyzed (1) before and after ON trials, (2) before and after OFF trials, and (3) the elevation of concentrations of cytokines during ON trials was compared with that of OFF trials.

Analyses resulting in statistical significance (p<0.05) or statistical tendency (0.05<p<0.1) are shown in the appropriate figures.

**Fig 5:**
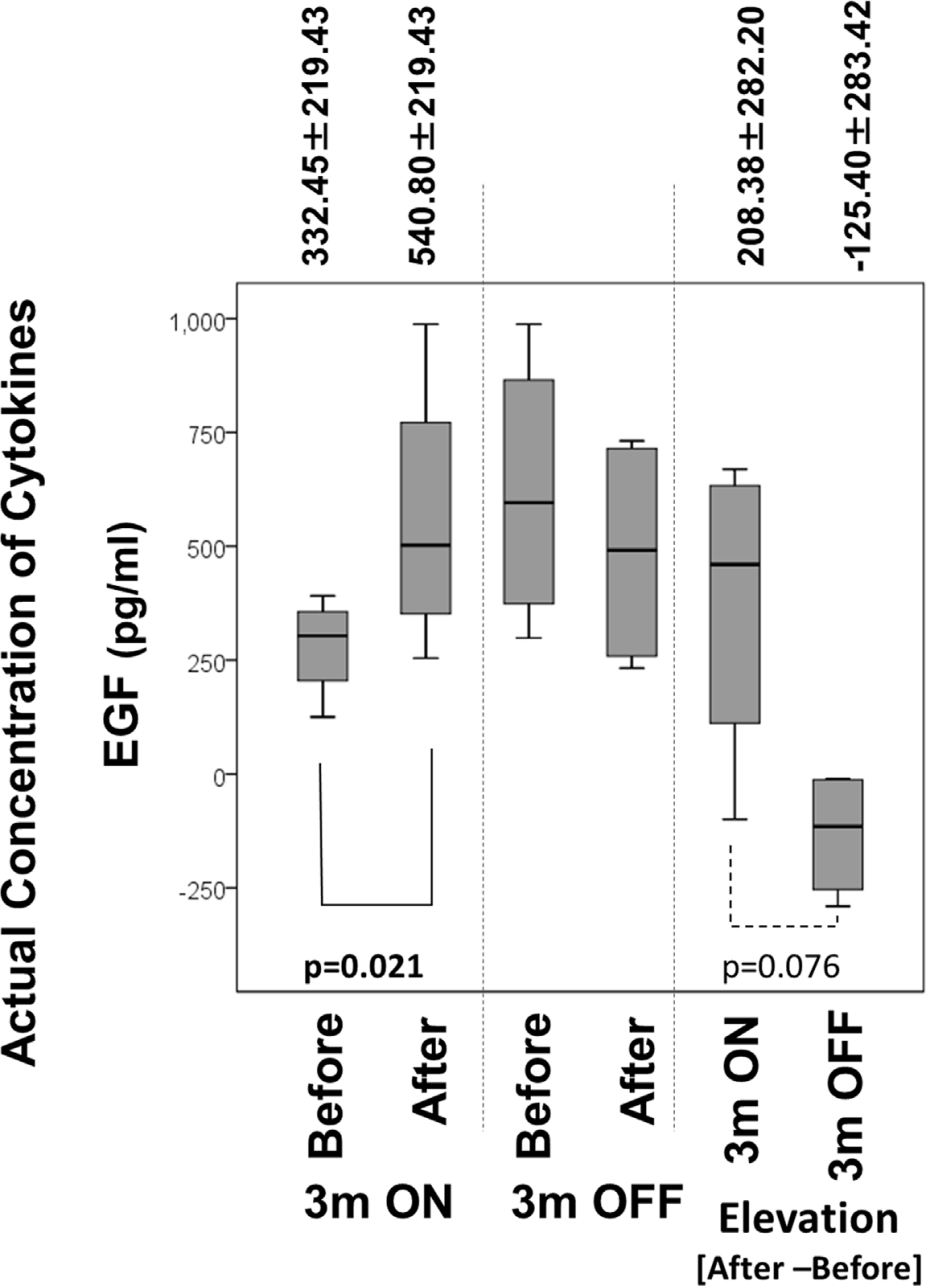
Changes of the actual concentrations of EGF in plasma during ON and OFF trials of NCPDIAC and comparison of the elevation/reduction of both cytokines between ON and OFF trials. The plasma concentration of EGF increased significantly during ON trials. In addition, comparison of the changes (elevation and reduction) of concentration of EGF between On and OFF trials showed a tendency of elevation in ON trials.

Regarding changes of actual concentrations, two cytokines, epidermal growth factor (EGF) showed alterations that were statistically significant or exhibited a tendency (Fig 5). The concentration of EGF was elevated significantly during ON trials and the elevation of ON trials was tended to be higher than that of OFF trials (the elevation of OFF trials actually exhibited a negative value, indicating that EGF concentrations during OFF trials were reduced).

#### 2. Changes of Log_10_ concentrations of cytokines

**Fig 6:**
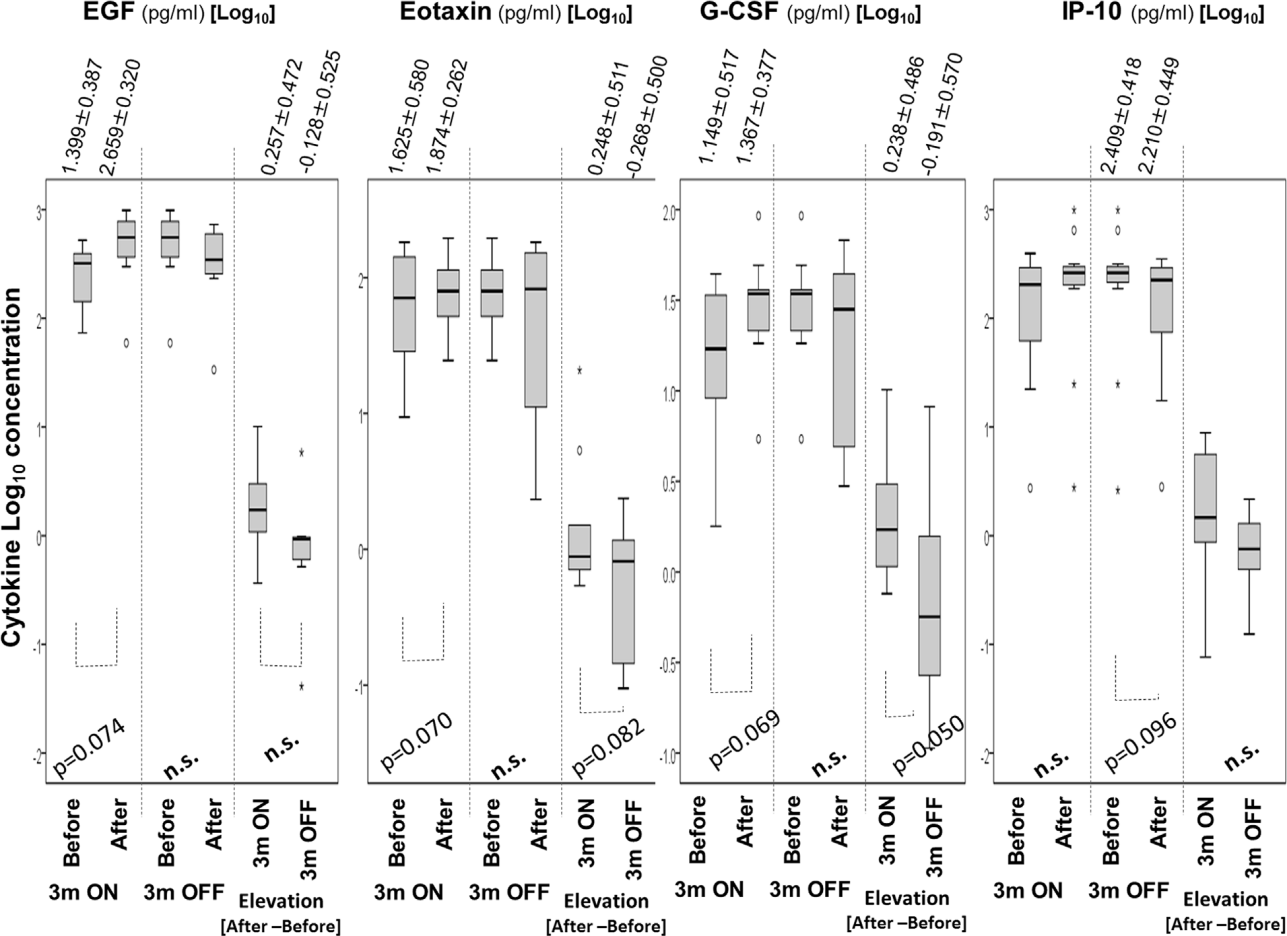
Changes of Log_10_ concentrations of EGF, Eotaxin, G-CSF and IP-10 in plasma during ON and OFF trials of NCPDIAC and comparison of the elevation/reduction of both cytokines between ON and OFF trials. The Log_10_ concentrations of EGF, Eotaxin and G-CSF tended to be increasing during ON trials, and comparison of changes (elevation or reduction) between ON and OFF trails showed a tendency of higher in ON trials. The IP-10 showed the tendency of reduction during OFF trials.

Fig 6 shows changes of Log_10_ concentrations of cytokines which showed statistical tendencies. EGF showed increasing tendency during 3 months ON. The log_10_ concentrations of Eotaxin and Granulocyte-Colony Stimulating Factor (G-CSF) during 3 months ON tended to be higher and elevation during 3 months OFF showed lower tendency than that of 3 months ON trials (actually, 3 months OFF trials showed less than 0 value meaning the Log_10_ concentration of Eotaxin and G-SCSF were reduced during OFF trails). In addition, the Log_10_ concentration of interferon-inducible protein 10 (IP-10) showed tendency of decrease during 3 months OFF trials.

#### 3. Changes of the ratio of Log_10_ concentrations of cytokines

Analysis of changes of the ratio of Log_10_ cytokine concentrations of EGF, IP-10, Eotaxin, G-CSF and interferon (IFN)α2 showed significant changes or tendencies of changes.

As shown in S2 Fig, ratio of Log_10_ concentration of EGF during 3 months ON trial showed tendency of increase. Those of IP-10 during 3 months OFF showed the tendency of decrease. In addition, the comparison of elevations during ON and OFF trials of IP-10 showed tendency to be lower in OFF trials.

As shown in S3 Fig, ratio of Log_10_ concentartion of Eotaxin during 3 months ON trial showed tendency of increase. In addition, comparison of elevation between ON and OFF trials tended to be lower in OFF trial in Eotaxin. G-CSF showed siginificant increase of Log_10_ ratio of concentration during ON time and the comparison of elevation between ON and OFF trials showed higher in ON than OFF trials. In addition, IFNα2 during ON trial showed tendency of increase in ratio of Log_10_ concentration.

The overall results of the 29 kinds of cytokines assayed revealed that EGF seemed to be elevated during NCPDIAC ON trials. NCPDIAC ON trials also seemed to induce elevation of Eotaxin and G-CSF. In addition, IP-10 was reduced by NCPDIAC OFF trials. All of these cytokines exhibited changes during NCPDIAC ON or OFF trials, even though the values were small.

### Formula detecting NCPDIAC ON or OFF periods

After analyzing the changes of concentrations of cytokines, attempts to generate a formula that will detect ON or OFF trials using several cytokine concentrations were performed using multi-regression analysis and stepwise multi-regression analysis. The elevation/reduction (after ON or OFF trial data minus values before the trial) of actual concentrations of cytokines were used in the analysis that showed a statistical significance or tendency by comparing values before and after the ON or OFF trials, or exhibited elevation/reduction of concentrations between ON and OFF trials for EGF, IP-10, Eotaxin, G-CSF, GM-CSF, IFNα2, IL-8 and MIP1α.

The multi-regression analysis extracted the three cytokines GM-CSF, EGF and IP-10 as forming the significant variable to detect ON (value of 1.0) or OFF (value of 0.0) trials. The formula designating the SUMICAS value is represented by “0.6082 - [GM-CSF] x 0.05426 + [EGF] x 0.00563]–[IP-10] x 0.00571”. This formula indicated that changes of the actual concentrations of EGF and IP-10 after three months indicated positively whether NCPDIAC was ON or OFF during the previous three months, while those of GM-CSF were negatively correlated.

Stepwise multi-regression analysis indicated that only the change of EGF concentration during three months was meaningful, and the other cytokines were therefore omitted from the analysis. As a consequence, the formula designating the SUMICAS_SW_ value is represented by “0.5434 + [EGF] x 0.00895”.

**Fig 7:**
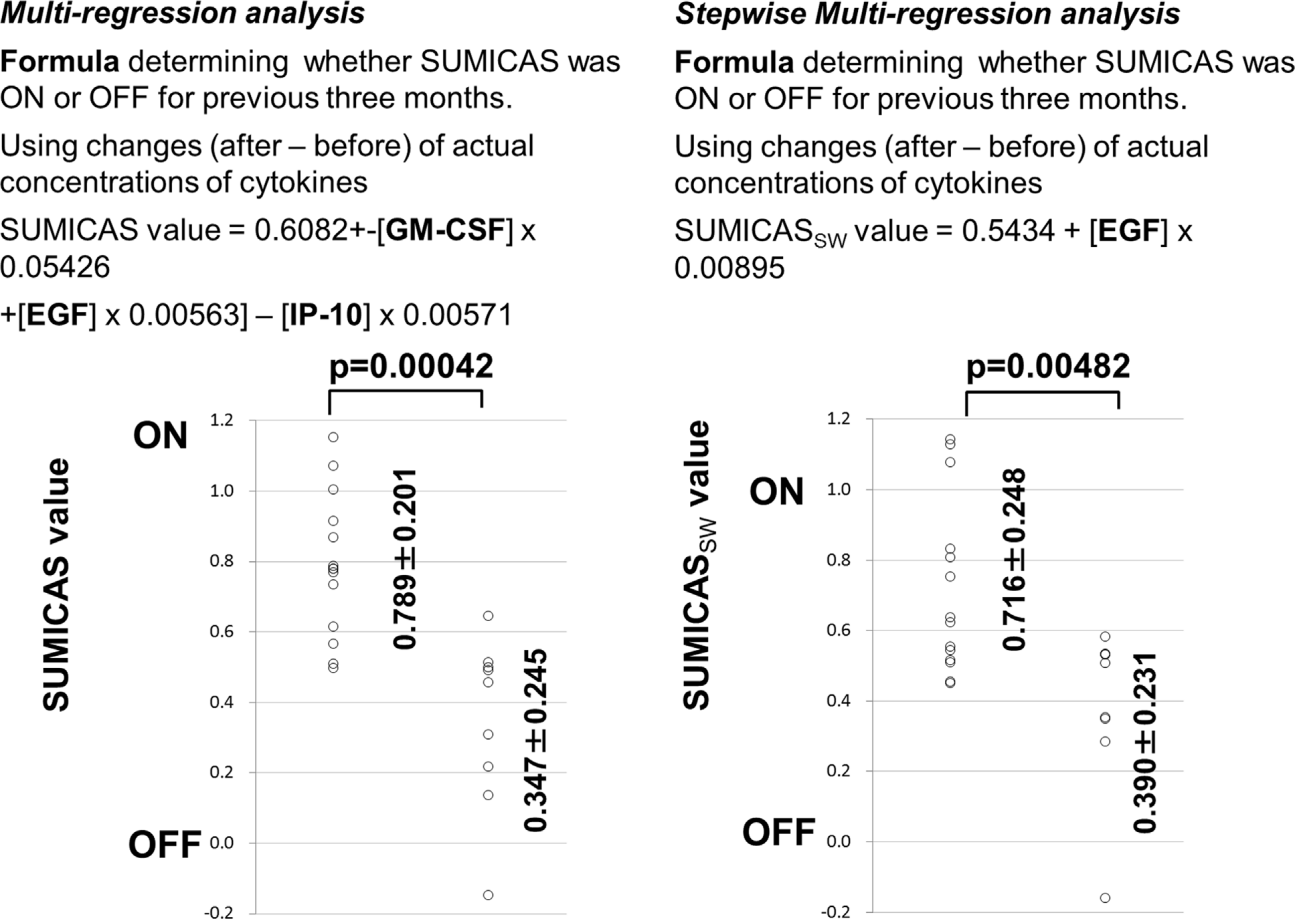
A multi-regression test (left panel) and stepwise multi-regression test (right panel) were performed using the changed actual concentrations of cytokines after ON or OFF trials relative to those before trials. These tests extracted cytokines that changed during ON or OFF trials and their values were applied as the variable items to detect ON or OFF trials when changes of cytokines showed a certain number. The number 1.0 was applied for ON trials and 0.0 was applied for OFF trials in both regression tests. Multi-regression analysis extracted the changed actual concentrations of EGF, GM-CSF and IP-10 as the significant variables with statistical significance. The formula to detect an ON trial is “0.6082 - [GM-CSF] x 0.05426 + [EGF] x 0.00563]–[IP-10] x 0.00571”. The stepwise analysis only extracted EGF from the eight cytokines tested. The formula is “0.5434 + [EGF] x 0.00895”. Significant differences were found with both formulas when used to detect whether NCPDIAC was ON or OFF during a three-month trial period using individual changes of after-minus-before trial values of the actual cytokine concentration from each volunteer.

Significant differences were found with both formulas when used to detect whether NCPDIAC was ON or OFF during a three-month trial period using individual changes of after-minus-before trial values of the actual cytokine concentration from each volunteer (Fig 7).

Our findings indicate that the change of EGF after a three-month stay in NCPDIAC is mostly affected by the condition determined by all of the 29 kinds of cytokines examined.

## Discussion

The indoor-air atmosphere is very important for human health because no one can live without staying in an indoor environment, which typically comprises a home, offices or public buildings. The development of disorders such as SBS and MCS prompts us to focus on the health effects of indoor air-conditions; however, medical science has unfortunately not discovered an adequate treatment for these disorders, and just avoiding exposure is the only advice available for these patients.

In accordance with these reflections, the development of living conditions that promote and enhance health is being considered along with the avoidance of various harmful chemicals. We have strengthened these developments by the establishment of NCPDIAC with analyses of the biological effects of short-term hour-long (2.5-hour stay) and mid-term week-long (two weeks of night stay) experiments. Results showed that hour-long stay trials induced a slight but significant increase of IL-2. The week-long stay trials then revealed enhancement of NK cell activity. These findings suggested that the slight increase of IL-2 during the night-time for two weeks caused accumulation of recurrent stimulation of NK cells and induced an increase of NK cell killing activity. We therefore thought that the NCPDIAC device needed to be tested in the indoor living environment of humans using stay experiments involving periods of months to over a year [16,17].

The results of our research indicate that NK cell activity was upregulated during stays involving NCPDAIC ON trials and downregulated during OFF trails. Of course, there are many factors that influence NK activity such as mental moods and stresses, nutrition status including the intake of probiotics such as lactic fermenting beverage, sleeping, exercise conditions, and age [24–26]. Even though these factors may change daily in the ordinary lives of individual volunteers, all blood sampling was performed at an adequate time (9 to 12 o’clock in the morning) and assayed in the same clinical examination testing company. The results from a total of 16 ON and 13 OFF trials may therefore suggest the definite alteration of NK cells caused by the NCPDIAC atmosphere. NCPDIAC may therefore promote and enhance human health by decreasing the prevalence of cancer and alleviating the symptoms of viral or bacterial infections such as influenza.

In addition to investigating NK activity, this study analyzed alterations of cytokines in regard to NCPDIAC. Results showed that EGF was elevated in NCPDIAC. Eotaxin and G-CSF were then also shown the tendency of increase in NCPDIAC ON trials. Moreover, IP-10 showed the tendency of reduction during OFF trials, but among the analyzed cytokines, the importance of EGF, and subsequently IP-10, was shown by multi- and stepwise regression analyses. EGF was only extracted by the stepwise method and detecting whether an ON or OFF trial was conducted could be made using the SUMICAS_SW_ formula and the value indicating how much the concentration was changed during the three-month trial period. NCPDIOAC had the largest effect on EGF among the various examined cytokines. Unfortunately, there is no investigation concerning how EGF influences NK cell activity. Regarding the results involving alterations of cytokines, it would be better to consider that these alterations were induced by NCPDIAC and that they represent another aspect of the immunological effects of NCPDIAC, independent from the enhancement of NK cell activity. As we reported previously [16,17], NK activity might be enhanced by recurrent and slight accumulation of the increase of IL-2. However, the concentration of IL-2 did not change following examination every three months in ON to OFF and OFF to ON trials of NCPDIAC. This is reasonable because the previous data indicated that the change of IL-2 in NCPDIAC was very slight, even if it was significant, and perhaps the up- and down-regulation occurred quickly for IL-2. The enhancement of NK activity was just observed in results outlining the accumulation of up- and down-regulation of IL-2.

A consideration of the alterations of cytokines such as EGF, Eotaxin and G-SCF shows that all of the changes of cytokines found in this study involved elevation after NCPDIAC ON trials, or significant differences between an elevation during the ON period and reduction in the OFF period. These results indicated that NCPDIAC resulted in a slight stimulation of the human immune system. However, it is important to note that none of the elevated cytokine values reached pathological levels.

EGF promotes cellular proliferation and differentiation [27–29]. The receptors for EGF are considered important concerning the development of cancer. The increased activity of receptors, as well as mutations of receptors, is known in lung, breast and other solitary tumors [30,31]. Basically, these receptor modifications activate EGF subsequent signaling pathways such as Ras and mitogen-activated protein (MAP) kinase pathways to induce continuous cellular proliferation [32,33]. On the other hand, EGF has recently been used in components for the treatment of intractable skin ulcers and burn ulcers utilizing its capacity to induce proliferation of skin epidermis [34,35]. Similar to these treatment materials, some cosmetics for anti-aging also include EGF [36,37]. Therefore, the slight and significant increase of EGF induced by NCPDIAC found in this study may be considered a kind of immune stimulation causing activation of epidermal functions, and may not adversely affect healthy individuals. It is possible that NCPDIAC can improve skin wounds or produce beneficial anti-aging effects. The fear is when individuals already possess some solitary tumors in which EGF receptors are activated. However, the increased level of EGF following NCPDIAC is slight, and the effects might not be persistent. The effect of NCPDIAC seemed to elevate basic EGF production to a slight degree. Taken together with the enhancement of NK activity, it would be reasonable to suggest that the slight increase of EGF does not influence cancer initiation and promotion even for cancers in which EGF receptors are activated [38,39]. Notwithstanding these considerations, it might be better to perform a year-long follow-up of individuals subjected to NCPDIAC.

Other cytokines that were altered by NCPDIAC such as Eotaxin and G-CSF were also tended to be elevated under these conditions, although the levels were slight and are not considered to produce a pathological status. We should consider the slight elevation of these cytokines as a response by individuals exposed to NCPDIAC and reflecting a slight immune stimulation status that will prevent infections and reduce the occurrence of cancers. However, it is not known how allergic reactions proceed in individuals exposed to NCPDIAC. The healthy volunteers recruited in this study did not show any newer allergic problems, except for one female who already had an allergy to some kinds of metals and continued to have the problem of metal allergy. However, all the specific IgE titers for 33 kinds of allergens did not change during any of the periods of NCPDIAC exposure among any of the volunteers. It cannot be concluded that NCPDIAC induces a worse reaction to allergic situations, or that it worsens allergies for individuals already possessing certain allergies.

In addition, certain cytokines which induce the pathological immune stimulation status such as allergy and autoimmune disorders such as IL-2, -4, -5, and IL-7 were not changed. Even they were not shown any tendencies of alterations. Thus, even though the cellular ad physiological mechanisms were not clarified in this NCPDIAC system, the immunosimulation seemed to be restricted to NK activity and not affected to T cells according to the analyses of cytokine changes. We considered that possibility of induction of pathological immunostimulation caused by living in NCPDIAC system seems to be very slight and within the safe level.

To summarize our study, NCPDIAC caused a slight but definite increase of IL-2 during short-term and mid- to long-term (weeks to months) exposure periods and induced NK cell killing activities due to the recurrent and accumulated elevation of IL-2, as well as a slight upregulation of immune status in individuals to result in protection against inflammation and the occurrence of cancers. Therefore, conditions created by the NCPDIAC device promote human health in the indoor environment and may be used in the home or office buildings to improve the health of occupants. It will be beneficial to conduct a year-long follow-up of healthy volunteers in the future by setting the NCPDIAC device to continuously ON and then examining the occurrence of various diseases, particularly allergies, infections and cancers.

## Supporting Information

S1 Fig The NK activities during the ON trials were tended to be elevated when the activity was assayed with a 10:1 or 20:1 Effector: Target cell ratio (ET ratio). In addition, actual NK activities assayed with a 10:1 or 20:1 ET ratio during OFF trial showed the decreasing tendency.
(TIF)

S2 Fig Changes of the ratio of Log_10_ concentrations of EGF and IP-10. The tended changes were found regarding an increase of EGF during ON trials and a decrease of IP-10 during OFF trials. The comparison of changes (elevation or reduction) between ON and OFF trials showed tendency of difference for IP-10.
(TIF)

S3 Fig Changes of the ratio of Log_10_ concentrations of Eotaxin, G-SCF and IFNα2. Significant changes were found in G-SCF during ON trials and comparison of elevation between On and OFF trials. The ratio of Log10 concentration of G-CSF was higher in ON trials than that of OFF trials (OFF trials showed less than 0 average means ratio was reduced), In addition, Eotaxin showed the tendency to be increasing during ON trials and comparison of elevation/reduction tended to be higher in ON trials. Regarding IFNα2, ON trials induced the tendency of increasing.
(TIF)
