# Supplementary figures and images for: Enhancement of NK Cell Cytotoxicity Induced by Long-Term Living in Negatively Charged-Particle Dominant Indoor Air-Conditions
Source: PLoS One. 2015 Jul 14;10(7):e0132373. doi: 10.1371/journal.pone.0132373 (PMC4501842; doi:10.1371/journal.pone.0132373)

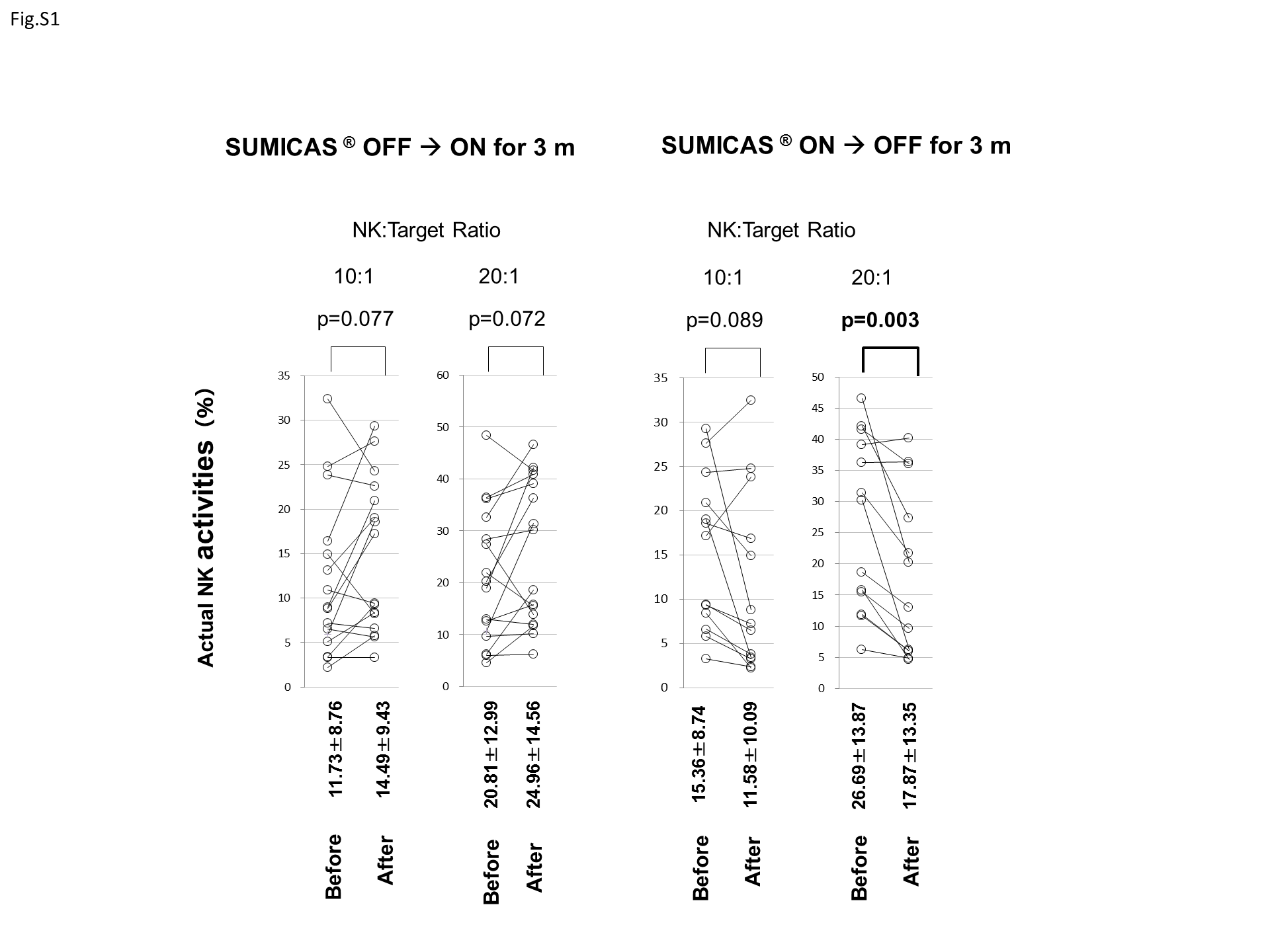

Supplement: S1 Fig — In addition, actual NK activities assayed with a 10:1 or 20:1 ET ratio during OFF trial showed the decreasing tendency. (TIF) [file pone.0132373.s001.tif]

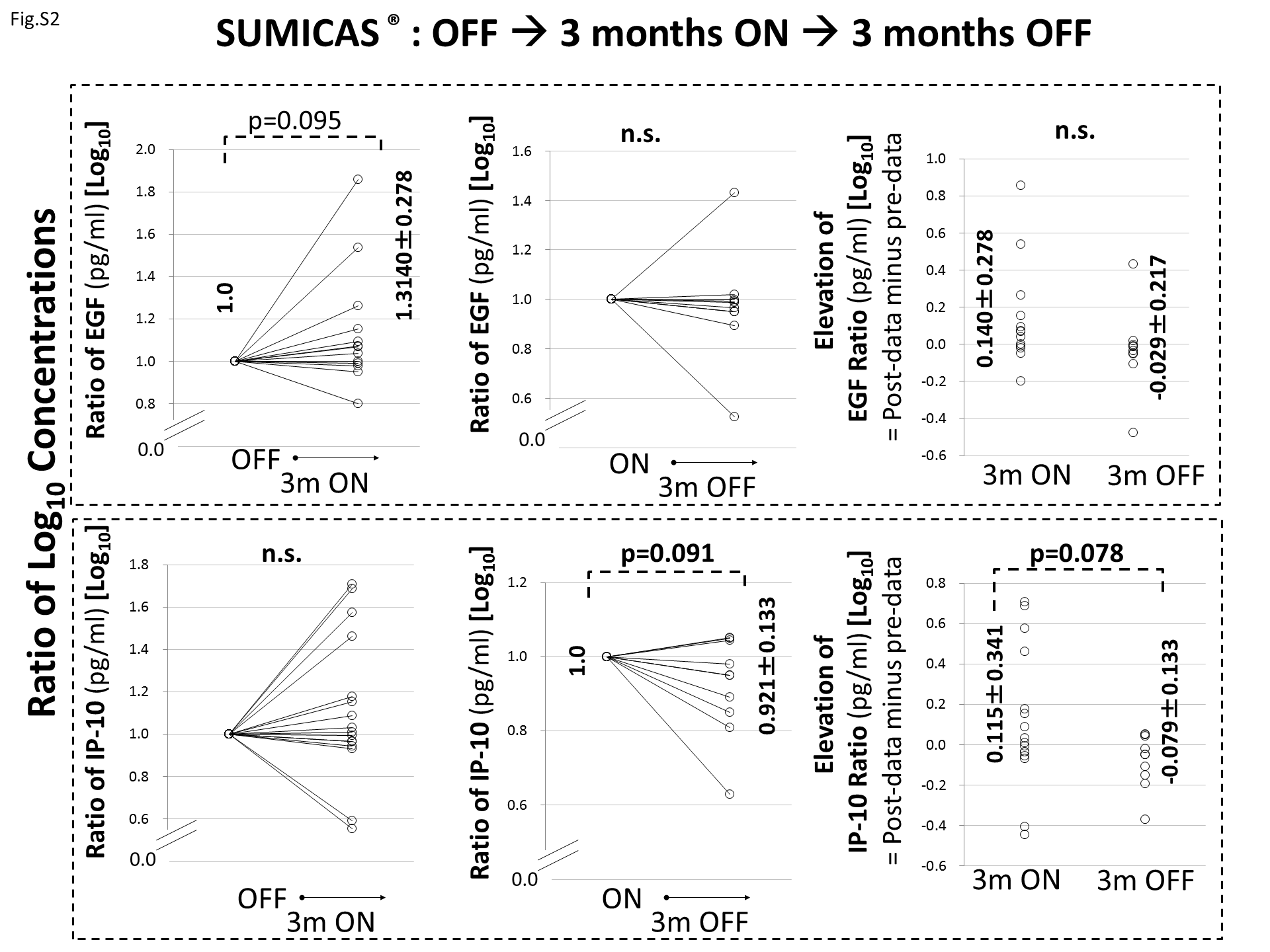

Supplement: S2 Fig — The tended changes were found regarding an increase of EGF during ON trials and a decrease of IP-10 during OFF trials. The comparison of changes (elevation or reduction) between ON and OFF trials showed tendency of difference for IP-10. (TIF) [file pone.0132373.s002.tif]

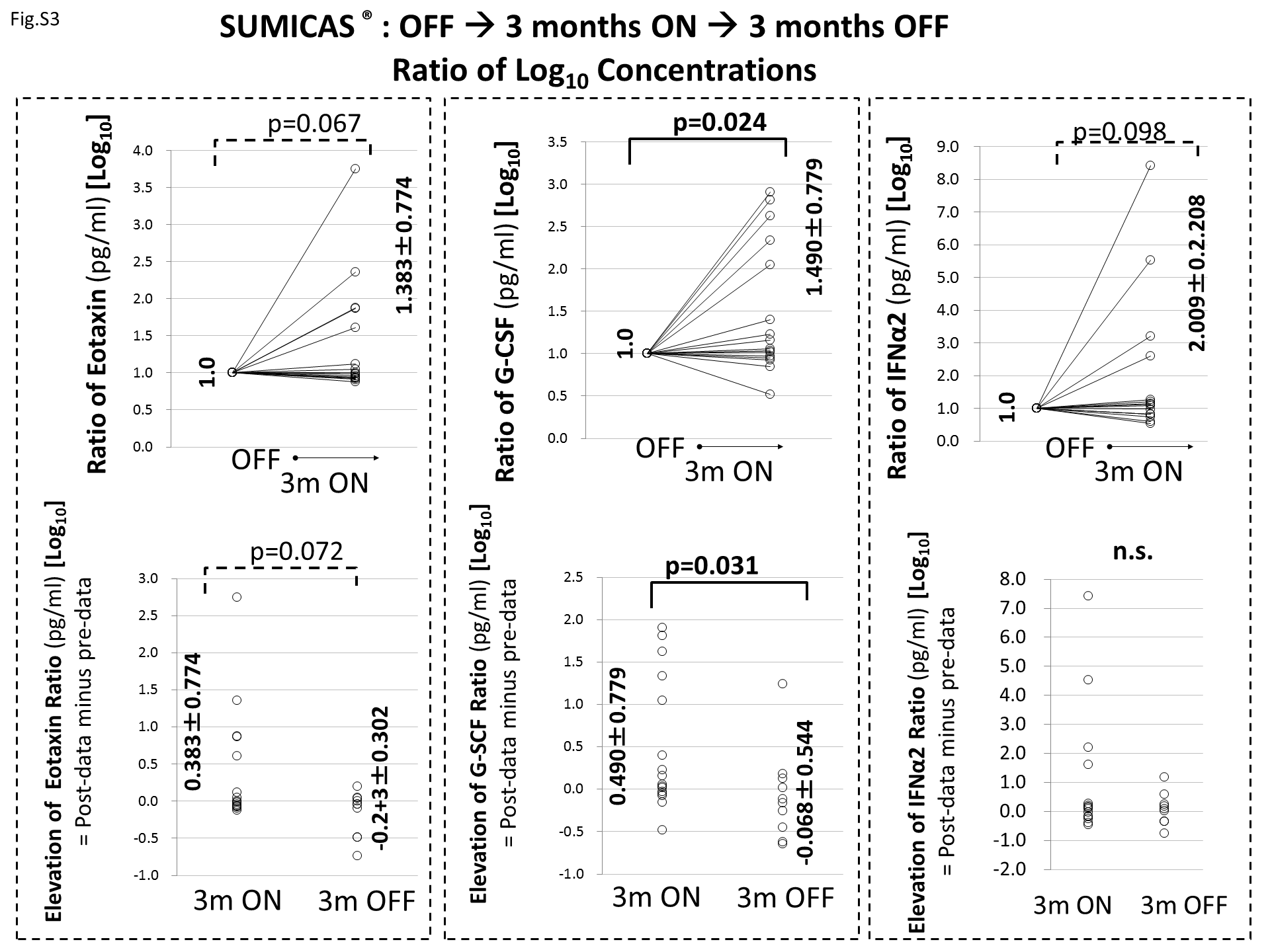

Supplement: S3 Fig — Significant changes were found in G-SCF during ON trials and comparison of elevation between On and OFF trials. The ratio of Log10 concentration of G-CSF was higher in ON trials than that of OFF trials (OFF trials showed less than 0 average means ratio was reduced), In addition, Eotaxin showed the tendency to be increasing during ON trials and comparison of elevation/reduction tended to be higher in ON trials. Regarding IFNα2, ON trials induced the tendency of increasing. (TIF) [file pone.0132373.s003.tif]
